# Decoding *Acorus verus*: Chloroplast Genome Insights into a Key Medicinal Resource from Guangxi, Southern China

**DOI:** 10.3390/genes17101197

**Published:** 2026-09-27

**Authors:** Junyu Chen, Yongxing Lai, Yinghan Dai, Meng Ni, Lijuan Song, Yuning Liang, Ziheng Xu, Minjie Jiang, Haibo Tang

**Affiliations:** 1 Institute of Traditional Chinese and Zhuang-Yao Ethnic Medicine, Guangxi University of Chinese Medicine, Nanning 530200, China; 2 School of Economics and Management, Guangxi Vocational University of Agriculture, Nanning 530007, China; gxulyx2008@126.com; 3 Guangxi Zhuang Autonomous Region Engineering Research Center of Graphene Biomedical Application Technology, Guangxi University of Chinese Medicine, Nanning 530200, China; 4 Guangxi Key Laboratory of Translational Medicine for Treating Infectious Diseases with Integrative Medicine, Guangxi University of Chinese Medicine, Nanning 530200, China; 5 Guangxi Health Commission Guangxi Key Laboratory of Molecular Biology of Preventive Medicine of Traditional Chinese Medicine, Guangxi University of Chinese Medicine, Nanning 530024, China

**Keywords:** *Acorus verus*, chloroplast genome, phylogeny, codon preference, repeat sequence

## Abstract

Background/Objectives: *Acorus verus* (*A. verus*), an important medicinal plant with increasing interest in genomic characterization, relies on genomic research to improve its germplasm and industrial applications. Methods: To fill the gap in chloroplast genome data for this species in Guangxi, China, this study sequenced and annotated its chloroplast genome. Results: The research analyzed structural features, codon usage, simple sequence repeats, Inverted Repeat Region boundaries, and phylogenetic relationships. The results indicated that the chloroplast genome of *A. verus* from Guangxi has a total length of 152,784 bp, exhibiting a typical cyclic tetrad structure. The GC content was 38.74%, with 129 annotated genes: 83 protein-coding, 38 tRNA, and 8 rRNA genes. Codon preference analysis identified 30 high-frequency codons, mostly ending in A/T (72.3%). There were 89 SSR sites, with 73% being single-nucleotide repeats favoring AT bases. Several variable regions were identified, including *psbM–trnD-GUC* in the Large Single-Copy Region (LSC) and *ndhF–rpl32* in the Small Single-Copy Region (SSC) suggesting their potential as candidate loci for future molecular identification and provenance studies. Phylogenetic analysis placed the Guangxi accession within the *A. verus* lineage, closely related to the Yunnan and Fujian accessions. Conclusions: This study provides the first chloroplast genome resource for *A. verus* from Guangxi and establishes a reference for accession authentication, comparative plastome analysis, germplasm evaluation, and sustainable utilization of this regional medicinal resource (PZ698195).

## 1. Introduction

*Acorus verus* (L.) Raf. (previously reported as *A. tatarinowii* Schott), a perennial herb belonging to the genus *Acorus* in the family *Acoraceae*, is utilized for medicinal purposes through its dried rhizome. It was first documented in “Shennong’s Classic of Materia Medica” [1] and is recognized as one of the oldest aromatic and orifice-opening medicinal materials in traditional Chinese medicine. Its industrial value chain encompasses various sectors, including health care, fine chemicals, and cultural consumption, establishing it as a high-value-added economic crop characterized by typical “green intellectual property” traits. The 2020 edition of the Chinese Pharmacopoeia notes its effects in opening orifices, eliminating phlegm, awakening the mind, enhancing intelligence, eliminating dampness, and stimulating appetite. Clinically, it is employed in the treatment of neurological disorders such as dementia and epilepsy, as well as cardiovascular, digestive, and respiratory system ailments [2].

Notably, as a classic orifice-opening and intelligence-promoting herb, *A. verus* occupies an irreplaceable position in numerous well-known traditional prescriptions: for example, it serves as the principal component in Changpu Yujin Tang for dispelling phlegm and opening orifices in the treatment of neuropsychiatric disorders such as tic disorder [3], in Ditang Tang for resolving phlegm and awakening the mind to treat dementia and stroke sequelae [4], and in paired combination with *Polygala tenuifolia* (Yuanzhi) for the prevention and treatment of dementia [5]. In Guangxi, a core genuine producing region, this herb has long been embedded in the medical systems of local Zhuang and Yao ethnic groups. It is a staple of classic Zhuang and Yao prescriptions formulated to awaken the mind, benefit intelligence, and resolve dampness [6], and remains widely dispensed across Guangxi to this day, particularly for conditions such as dementia, stroke sequelae, pediatric tic disorder, depression, and insomnia [5,7,8]. The prevalence of neurodegenerative and cerebrovascular disorders in Guangxi has risen alongside population aging [9,10], and *Acorus*-containing formulas are among the most frequently prescribed remedies for managing these conditions in the region [11]. These applications further consolidate its distinctive clinical value in Guangxi.

Contemporary chemical and pharmacological research has revealed that *A. verus* is abundant in volatile oils, terpenoids, and other active components, and β-asarone and α-asarone are the primary characteristic components that exhibit neuroprotective and memory-enhancing properties, indicating its promising potential for the treatment of Alzheimer’s disease [12]. In recent years, the implementation of the “Healthy China” strategy and the rapid growth of the traditional Chinese medicine industry have led to a sustained increase in market demand for *Acorus calamus*. This demand has facilitated a shift from traditional wild harvesting methods to industrialized practices, such as wild-like planting in forested areas and agroforestry operations. *A. verus* serves as a representative economic medicinal plant within forest ecosystems, benefiting from the resource endowment that allows it to “not occupy prime farmland while generating income from both forestry and medicine.”

Guangxi lies in the southern subtropical zone and contains extensive karst landscapes. Carbonate bedrock, heterogeneous microhabitats, strong vertical climatic gradients, and complex hydrothermal conditions can shape the growth and metabolite accumulation of karst-adapted medicinal plants [13,14]. A shade-tolerant, moisture-loving rhizomatous herb such as *A. verus* may benefit from the humid, shaded, and well-drained conditions of karst forest understories and rock crevices. These ecological features provide a plausible basis for regional differences in growth and medicinal quality, but their effects on asarone content or clinical quality were not tested in this chloroplast study. *A. verus* was included in the Guangxi Directory of Authentic Medicinal Materials in 2023 [15]. At the sector level, a regional revitalization plan set a target of RMB 18 billion for medicinal-material cultivation and breeding by 2025 [16]. Because publicly available policy documents do not report a standardized province-wide annual output value for *A. verus* alone, this study treats its daodi status as a regional production and quality context rather than assigning an unsupported species-specific economic constant. Molecular traceability is consequently important for linking local germplasm to future quality and production studies.

Chloroplasts, which are semi-autonomous organelles exclusive to plants, serve as the sites of photosynthesis. Their genomes typically exhibit a conserved circular quadripartite structure and a moderate evolutionary rate, making them an indispensable tool for resolving phylogenetic relationships, uncovering evolutionary trajectories, and developing molecular markers for germplasm identification [17,18,19]. However, recent organellar comparative genomics has revealed that plant plastomes are not entirely static; they undergo dynamic lineage-specific variations, such as inverted repeat (IR) expansion/contraction, structural rearrangements, and localized mutational hotspots driven by distinct DNA repair and replication machineries [19,20]. Currently, published chloroplast genomic data for the genus *Acorus* remain limited, primarily encompassing *A. verus* from Xishuangbanna, Yunnan [21], *A. verus* from Fujian [22], *Acorus* calamus L. [23], *Acorus shannai* [24], and *Acorus gramineus* Soland. [25]. Notably, Guangxi, recognized as the core distribution area and a significant native production region for *A. verus*, lacks relevant genomic reports, restricting comparative analyses of plastome diversity and structural evolution across different populations.

This study employed high-throughput sequencing and assembly techniques to analyze the chloroplast genome of *A. verus* from Guangxi. It systematically examined various genomic structural characteristics, including genome size, GC content, the expansion and contraction of the IR boundary, the distribution patterns of simple repeat sequences, and codon usage preferences. The findings will address the existing gap in chloroplast genome resources for *A. verus* in the Guangxi region, enhance the chloroplast genome database for the *Acorus* genus, and provide genomic resources for future studies on genetic diversity, taxonomy, and plastome evolution within the genus *Acorus*. Additionally, this research will support the conservation, development, and utilization of germplasm resources.

## 2. Materials and Methods

### 2.1. Materials

Fresh leaves of *A. verus* were collected by Prof. Tang Haibo at the base of Daming Mountain in the Wuming District of Nanning City, Guangxi (23°24′ N latitude, 108°20′ E longitude, approximately 200 m above sea level) in January 2025. The identification was conducted by the Zhuang and Yao Medicine Laboratory at Guangxi University of Chinese Medicine. The diagnostic basis for *A. verus Acorus verus* includes the following typical features. The plant is caespitose. The rhizome is aromatic, pale brown, 2–5 mm in diameter, with internodes 3–5 mm long, and densely branched in the upper part. The leaves are thin, dark green, linear, 20–30 cm long, conduplicate at the base, flattened below the middle, gradually narrowed at the apex, lacking a midrib, and with numerous slightly raised parallel veins. The leaves are sessile; at the base, they bear membranous sheaths on both sides, which are about 5 mm wide, extending upward to the middle of the leaf, gradually narrowing, and eventually deciduous.

### 2.2. Extraction of Chloroplast Genomic DNA

Fresh leaves of *A. verus* collected from Guangxi are placed in sampling bags, rapidly frozen in liquid nitrogen, and stored at −80 °C for subsequent DNA extraction. The samples on dry ice were transported to Shanghai Tianhao Biotechnology Co., Ltd. (Building 9, No. 787 Kangqiao Road, Pudong New Area, Shanghai, China)for DNA extraction and library construction. Genomic DNA extraction was performed using a modified CTAB method [26].

### 2.3. DNA Library Construction and Sequencing

After the DNA test was qualified, the genomic DNA of the sample was fragmented by the Covaris ultrasonic random fragmentation method. Then, it was purified, the sequencing connector was joined, the end repair was carried out, an A-tail was added to the 3′ end, PCR amplification was performed, and the library preparation work was completed. The libraries were inspected again. The libraries that passed the quality inspection were sequenced using the Illumina HiSeq 2500 high-throughput sequencing platform (Illumina, San Diego, CA, USA), and the sequencing part was completed by Shanghai Tianhao Biotechnology Co., Ltd. (Shanghai, China). Sequencing quality was evaluated based on the generated raw reads and filtered clean reads. The sequencing statistics, including total reads, bases generated, Q20 and Q30 quality scores, and GC content before and after filtering, are summarized in Supplementary Table S1.

### 2.4. Chloroplast Genome Assembly

Following the acquisition of raw reads through sequencing, a quality assessment was performed using FastQC [27]. Subsequently, quality control and filtering of the raw reads were executed with Trimmomatic v.0.33 [28] to yield high-quality, effective reads. Assembly graphs were visualized using Bandage [29] after assembly to confirm the circular structure of the plastome. The chloroplast genome was assembled de novo using GetOrganelle v.1.7.7 [30], and the published plastome of *A. verus* (MN536753) was used as a reference for assembly validation and annotation. Automatic annotation was carried out using CPGAVAS2 [31]. The results of the automatic annotation were imported into Geneious Prime v.11.0.1, where the Repeat Finder plugin was utilized to identify the reverse repeat regions. Finally, gene boundaries were manually inspected and corrected based on comparisons with published *Acorus* plastomes.

### 2.5. Chloroplast Genome Analysis

The chloroplast genomes of seven verified *Acorus* accessions representing five accepted taxa were selected for comparative analysis. Taxonomic identities and accession information were manually checked against GenBank records and the Plants of the World Online (POWO) database before downstream analyses.

#### 2.5.1. Codon Usage Analysis

All protein-coding genes (coding sequences, CDSs) of the chloroplast genome were extracted, and those with a sequence length of at least 300 bp were selected for analysis. The codon composition of each CDS was calculated using CodonW v.1.4.2 [32] (http://codonw.sourceforge.net/, accessed on 28 April 2026), and the relative synonymous codon usage (RSCU) for each codon was statistically analyzed [33]. The RSCU value represents the ratio of the actual usage frequency of a codon to the expected uniform usage frequency of synonymous codons, thereby reflecting codon usage preferences. The criteria for interpretation are as follows: an RSCU value greater than 1 indicates that the codon is frequently used and is considered a preferred codon; an RSCU value equal to 1 suggests no usage preference; and an RSCU value less than 1 indicates that the codon is used less frequently.

#### 2.5.2. SSR Analysis

Simple repetitive sequences in the chloroplast genome of *A. verus* from Guangxi were identified using the online MISA v2.1 [34] (https://webblast.ipk-gatersleben.de/misa/, accessed on 28 April 2026). The detection parameters for each type of simple sequence repeat (SSR) were established as follows: the minimum repeat counts for single nucleotides, dinucleotides, trinucleotides, tetranucleotides, pentanucleotides, and hexanucleotides were set to 10, 5, 4, 3, 3, and 3, respectively.

#### 2.5.3. Boundary Analysis of IR/SC

The boundary visualization analysis of chloroplast genomes from seven plastomes representing five *Acorus* taxa within the genus *Acorus*, originating from diverse locations, was performed using CPJSdraw software [35]. This analysis involved a comparison of the Large Single-Copy Region (LSC), Small Single-Copy Region (SSC), and Inverted Repeat a (IRa) across each genome. The study focused on the positional differences of the boundaries among the four regions (IRa) and Inverted Repeat b (IRb), with particular attention given to the expansion and contraction of the inverted repeat b (IR). Additionally, a statistical analysis was conducted to categorize the types and distributions of genes located in the boundary regions.

#### 2.5.4. Sequence Similarity Analysis

The mVISTA online platform [36] (https://genome.lbl.gov/vista/mvista/submit.shtml, accessed on 28 April 2026) and the Shuffle—LAGAN global alignment model were used to analyze the collinearity of the chloroplast genomes among Acorus species. The Guangxi *A. verus* plastome (PZ698195) was used as the reference sequence. This approach allows for an assessment of the overall structural variation within the chloroplast genome of the genus *Acorus*.

#### 2.5.5. Nucleotide Diversity Analysis

To quantitatively evaluate sequence divergence among the seven *Acorus* plastomes, all plastome sequences were first standardized to the same orientation and circular starting position according to the Guangxi plastome (*A. verus* PZ698195). Multiple sequence alignment was performed using MAFFT v7.107 [37] with default parameters. The aligned plastome sequences were subsequently imported into DnaSP v6 [38] for nucleotide diversity (π) analysis using a sliding-window approach with a window length of 600 bp and a step size of 200 bp. Regions showing elevated nucleotide diversity were identified according to the genomic coordinates and annotation information of the Guangxi plastome.

#### 2.5.6. Phylogenetic Analysis

A phylogenetic study was conducted based on the 7 plastomes from the genus *Acorus*, and *Kaempferia rotunda* L. was reserved as the outgroup [39]. Protein-coding sequences (CDSs) of chloroplast genomes were aligned with MAFFT v.7.107 [37], employing default parameters. The phylogenetic tree was constructed using two methods: Maximum Likelihood (ML) and Bayesian Inference (BI). The ML tree was generated on the PhyloSuite platform [40] utilizing IQ-TREE v.2.1.3 [41]. The best-fit model TVM + F + G4 was inferred by ModelFinder [42] based on the Bayesian Information Criterion (BIC), with the ultra-bootstrap value set to 1,000,000. The BI tree was constructed using MrBayes v.3.2.6 [43]. Similarly, the best-fit model (GTR + F + G4) was inferred by ModelFinder according to BIC, and the Markov Chain Monte Carlo (MCMC) algorithm was executed for a total of 2,000,000 generations. Upon completion, the average standard deviation of the separation frequency was assessed; a value less than 0.01 indicated convergence. The first 25% of “burn-in” samples were discarded, and the evolutionary tree was constructed with the remaining data. If the value was greater than or equal to 0.01, the process was repeated until convergence was achieved.

## 3. Results

### 3.1. Basic Characteristics of the Chloroplast Genome of A. verus in the Guangxi Region

The chloroplast genome of *A. verus* from the Guangxi region exhibits a typical tetrad structure, with a total length of 152,784 bp and an overall GC content of 38.74%. This genome comprises a large single-copy region (LSC), a small single-copy region (SSC), and a pair of inverted repeat regions (IRa and IRb), measuring 83,056 bp, 18,240 bp, and 25,744 bp, respectively (Figure 1). Annotation results indicate that the chloroplast genome of *A. verus* in Guangxi contains a total of 129 functional genes (Table 1), which can be categorized into four major functional groups: 45 genes associated with photosynthesis, 74 genes involved in self-replication, 4 additional functional genes, and 5 genes of unknown function. Among these, 15 genes contain a single intron, specifically *petB*, *petD*, *atpF*, *ndhA*, *ndhB* (two copies), *rpoC1*, *rpl16*, *rpl2* (two copies), *rps16*, *trnA-UGC* (two copies), *trnI-GAU* (one copy), *trnK-UUU*, *trnL-UAA*, *trnS-CGA*, and *trnV-UAC*. Additionally, three genes possess two introns: *rps12* (two copies), *clpP*, and *ycf3*. Furthermore, 17 genes are duplicated, including *ndhB*, *rpl2*, *rpl23*, *rps7*, *rrn16S*, *rrn23S*, *rrn4.5S*, *rrn5S*, *trnA-UGC*, *trnH-GUG*, *trnI-GAU*, *trnL-CAA*, *trnN-GUU*, *trnR-ACG*, and *trnV-GAC*, while *trnM-CAU* is duplicated four times.

### 3.2. Complete Chloroplast Genome Characteristics of Acorus Species

Seven complete chloroplast genomes of five accepted taxa within the genus *Acorus*, including the newly sequenced Guangxi accession of *A. verus* PZ698195, were compared with previously published plastomes (Table 2). After verification of accession identities and taxonomic names, all comparative analyses were conducted using independent chloroplast genome sequences. The comparative analysis reveals that the complete chloroplast genome sizes within the genus *Acorus* range from 152,784 bp (*A. verus* PZ698195) to 153,821 bp (*A*. *calamus*), with *Acorus* Guangxi exhibiting the shortest total genome length among the five species. Within the tetrad regions, *A. verus* from the Guangxi region has the shortest LSC region (83,056 bp) and IR region (25,744 bp), while the SSC region (18,240 bp) is consistent with that of *A. verus* from Fujian (MT755635). The *A. verus* from Guangxi is positioned at the intermediate level within the genus, with a sequence length of 18,232–18,273 bp. Its GC content is 38.74%, the highest among the five species examined. In terms of genetic composition, Guangxi *A. verus* has a total of 129 annotated genes. The number of tRNAs (38) and rRNAs (8) is consistent across all species, indicating a high level of conservation for these two types of non-coding RNA genes within the genus *Acorus* (Table 3).

### 3.3. Codon Preference Analysis

The codon usage frequency of 57 protein-coding genes in the chloroplast genome of *A*. *verus* from Guangxi was statistically analyzed. A total of 23,799 codons were encoded. The results indicated a significant preference for specific codons. All protein-coding genes in the chloroplast genome of *A. verus* PZ698195 encode all 20 standard amino acids. Among these, leucine (Leu) exhibited the highest usage frequency at 10.55% (2510), while cysteine (Cys) displayed the lowest frequency at only 1.12% (266) (Figure 2, Table 4). The preferred codons for leucine were UUA (RSCU = 1.79), CUU (RSCU = 1.30), and UUG (RSCU = 1.23). Additionally, AUU (RSCU = 1.38) for isoleucine and UCU (RSCU = 1.63) for serine also showed relatively high RSCU values. Most preferred codons showed a strong preference for A/U-ending codons. The RSCU value of the start codon AUG was 1.0. All three stop codons (UAA, UAG, and UGA) were detected, with UAA showing the highest RSCU value (1.39). Overall, 31 codons exhibited RSCU values greater than 1, whereas 31 codons showed RSCU values lower than 1. Most overrepresented codons terminated with A or U, whereas underrepresented codons were mainly C/G-ending. The proportion of A/T nucleotides at the third codon position was 72.3%, indicating a clear A/T bias in the codon usage pattern of the chloroplast genome of *A. verus* PZ698195.

### 3.4. Analysis of SSR Repeat Sequence Types

The simple sequence repeats (SSRs) in the chloroplast genome of *Acorus verus* from Guangxi were identified and statistically analyzed using MISA (https://webblast.ipk-gatersleben.de/misa/, accessed on 28 April 2026), resulting in the detection of 89 SSR loci (Figure 3A). Among the various SSRs, single-nucleotide repeats are the most prevalent, totaling 65 and comprising 73% of the overall SSR count. This type represents the predominant repeat category in the chloroplast genome, and this distribution aligns with the characteristics observed in the chloroplast genomes of most angiosperms. Trinucleotide repeats rank second, with a total of 9, accounting for 10.1%. Additionally, there are 6 dinucleotide repeats and 6 tetranucleotide repeats, each constituting 6.7%. Two pentanucleotide repeats account for 2.2%, while hexanucleotide repeats are the least common, with only one identified (Figure 3A).

In terms of motif composition (Figure 3B), the A/T motif is the predominant type among single-nucleotide repeats, accounting for 63 occurrences, while only 2 C/G types are present. This observation indicates a significant AT preference in the simple sequence repeats (SSRs) of this chloroplast genome, which correlates with the overall high AT content characteristic of chloroplast genomes. Such a preference is commonly observed in the chloroplast genomes of angiosperms. Among polynucleotide repeats, the dinucleotide repeat motif AT/AT exhibits the highest frequency of occurrence, with 6 instances, making it the dominant motif within this category. In contrast, trinucleotide to hexanucleotide repeats display a relatively high diversity in base composition, including the following: AAG/CTT (4), AAAT/ATTT (3), AAT/ATT (2), ATCC/ATGG (2), and ATC/ATG (2), as well as one each of AAC/GTT, AAGC/CTTG, AAGGG/CCCTT, AATT/AATT, and AGAGAT/ATCTCT (Figure 3B).

### 3.5. IR Region Boundary Analysis

The IR boundary regions of seven *Acorus* plastomes were compared (Figure 4). The findings indicated that the total length of the chloroplast genome for one variant of the seven *Acorus* plastomes representing five accepted taxa exhibited slight variation (152,784–153,821 bp), accompanied by differing degrees of contraction and expansion in each boundary region. Notably, the length variation of the IR region ranged from 25,744 to 26,025 bp. At the JLB/IRb boundary, the *rps19* genes across all five species spanned both the LSC and IRb regions. Among these, the length of the *rps19* gene in *A*. *verus* from Guangxi was relatively short, measuring only 25 bp within the IRb. Additionally, the *trnH* gene displayed a similar expansion length in the IRb, ranging from 248 to 253 bp. At the SSC/IRb boundary, both the *trnN* and *ndhF* genes are entirely situated within the IRb and SSC regions, without crossing the boundary. Notably, the *trnN* gene of *A. americanus* is positioned the farthest from the JSB boundary, at a distance of 1843 bp. Conversely, at the IRa/SSC boundary, the *ycf1* gene spans both the IRa and SSC regions. Within this context, the length of *ycf1* in the IRa region is the shortest, measuring 644 bp, which is approximately half the length observed in other species, ranging from 1172 to 1241 bp. At the IRa/LSC boundary, the *trnH* genes of *A. verus* from Guangxi, *A. verus* from Fujian, *A. gramineus*, and *A. shannai* are all located within the IRa region. Additionally, the *rps19* gene of *A. calamus* and *A. calamus var. americanus* extends to the JLA boundary. On the LSC side, the *psbA* genes of the five species are entirely contained within the LSC region and do not cross the JLA boundary.

### 3.6. Chloroplast Genome Alignment Analysis

Genome-wide homology comparisons among one variety of five species within the genus *Acorus* were performed using mVISTA (Figure 5). The findings indicate that the chloroplast genome structure of *Acorus* is highly conserved, with the rRNA gene clusters (*rrn16S*, *trnI-GAU*, *trnA-UGC*, *rrn23S*, *rrn4.5S*, and *rrn5S*) in the inverted repeat (IR) region arranged in reverse symmetry. Species within this genus exhibit consistency in gene content, arrangement, sequence, and transcriptional direction. No significant gene rearrangements, large fragment inversions, or gene losses were observed. In terms of sequence similarity, the overall conservation of the protein-coding region is notably high, with most regions approaching 100%. In contrast, the similarity curves of non-coding regions, including gene intervals and introns, exhibit significant concavity at multiple locations. These include the non-coding regions downstream of the *ndhF* gene, near the *ycf1* gene, between *trnK* and *rps16*, between *atpH* and *atpI*, and between *trnL* and *trnF*. This pattern indicates a concentration of sequence differences between *A*. *verus* from Guangxi and other species within the *Acorus* genus. The variable signals were distributed unevenly across the LSC and SSC regions, including spacers near *psbM–trnD-GUC*, *trnT-UGU–trnL-UAA* and *ndhF–rpl32*, while most IR sequences remained comparatively conserved.

To further quantify sequence divergence among the seven *Acorus* plastomes, nucleotide diversity (π) was evaluated using a sliding-window analysis (Figure 6). The mean nucleotide diversity was 0.0072, with a maximum π value of 0.0679. The strongest peak occurred in *psbM–trnD-GUC* in the LSC (π = 0.0679), followed by *ndhF–rpl32* in the SSC (π = 0.0438), *ndhF* (π = 0.0360), *trnT-UGU–trnL-UAA* (π = 0.0342), and the *atpF* intron (π = 0.0309). The IR regions were generally more conserved, although *ycf2* showed a lower peak of π = 0.0241. These hotspots were used as candidate regions for the marker-oriented interpretation in the Discussion section.

### 3.7. Phylogenetic Results

The ML and BI analyses produced identical topologies (Figure 7). Using *Kaempferia* rotunda as the outgroup, all sampled *Acorus* plastomes formed a strongly supported monophyletic clade (BS/PP = 100/1). Within *Acorus*, the *A. americanus* and *A. calamus* accessions formed the basal lineage. The remaining taxa formed a second major lineage, within which *A. gramineus* and *A. shannai* showed distinct relationships. The Guangxi plastome generated in this study was placed within the *A. verus* lineage and was sister to the previously reported Yunnan accession MN536753 and Fujian accession MT755635 with strong support (BS/PP = 100/1), confirming its close phylogenetic affinity with other *A. verus* plastomes.

## 4. Discussion

The chloroplast genome of *A*. *verus* from Guangxi, assembled in this study, has a total length of 152,784 bp and exhibits a typical cyclic tetrad structure. This characteristic is commonly observed in the chloroplast genomes of most terrestrial plants and is highly conserved among angiosperms [44]. The Guangxi plastome falls within the conserved genomic framework of Acorus, yet it is not identical to the published Yunnan and Fujian *A. verus* accessions. The Guangxi genome is 152,784 bp, compared with 153,296 bp for Yunnan (MN536753/NC_045294) and 153,453 bp for Fujian (MT755635) [21,22]. It also has the highest GC content among these three accessions (38.74%) and the fewest annotated genes (129), whereas both comparison accessions have 132 genes. The size difference is concentrated mainly in the shorter Guangxi LSC and IR regions. The SSC is 18,240 bp in both Guangxi and Yunnan and 18,232 bp in Fujian. These contrasts support the value of adding a Guangxi reference, but they describe accession-level differences rather than established population-level divergence. The number of tRNA and rRNA genes is identical to that of the other five species (Table 2), indicating strong intrageneric conservation in the chloroplast genome of Acorus at both the base composition and non-coding RNA levels. Despite the overall structural conservation, the chloroplast genome of *A*. *verus* from Guangxi also demonstrates a specific preference for codon usage at the sequence composition level.

Codon usage bias reflects nucleotide composition and evolutionary constraints [45]. This study found that the third base of the chloroplast codons in *A. verus* from Guangxi predominantly ends with A or T. Among a total of 25,219 codons, the majority of the 30 preferred codons with RSCU > 1 concluded with A or T, resulting in an average proportion of A/T at the third base of 72.3% (Table 1). The accumulation of GC→AT replacement mutations is closely associated with replication slippage and the specific efficiency of the organellar DNA repair system, which plays a pivotal role in retaining and fixing organellar nucleotide substitutions across seed plants [19,20]. The findings regarding *Acorus* species are consistent with previous reports [21,23], suggesting that the chloroplast genomes within the *Acorus* genus exhibit a notable degree of conservation in their codon usage strategies. Additionally, the distribution pattern of simple repetitive sequences, as another significant source of genomic variation, further underscores this conservation.

This study identified a total of 89 SSR loci in the chloroplasts of *A. verus* from Guangxi. Among these, single-nucleotide repeats were predominant, comprising 65 loci, or 73%, primarily consisting of A/T motifs (Figure 3A). Both the number and proportion of these repeats fall within the previously reported variation range for *A. calamus* and related monocotyledons [21]. This finding aligns with the general patterns of SSRs in the chloroplasts of angiosperms [46]. This phenomenon may be attributed to the simplicity of the chloroplast DNA repair mechanism, which facilitates the accumulation of single-base sliding mutations [47]. In contrast to the numerous but mutation-prone single-nucleotide repeats, polynucleotide repeats typically exhibit greater phylogenetic conservation [48]. The polynucleotide repeats in *A. verus*, such as the tetanucleotide AAAT/ATTT and the specific hexanucleotide AGAGAT/ATCTCT, demonstrate significant phylogenetic conservation and may serve as potential candidate sites for specific DNA barcodes.

The contraction and expansion of the boundary between the inverted repeat (IR) region and the single-copy region of the chloroplast genome significantly contribute to the variations in genome size among species [49]. The total length of the chloroplast genome and the length of the IR region in *A. verus* from Guangxi, as well as its related species, exhibit minimal differences. At the JLB/IRb boundary, the *rps19* gene in *Acorus verus* from Guangxi is located only 25 bp within the IRb, which indicates the least degree of expansion, representing a distinct contraction compared to the 87–93 bp expansion observed in *A. calamus* and *A. gramineus*. The expansion length of the *trnH* gene into the IRb region is remarkably consistent across the genus, ranging from 248 to 253 bp. This boundary structure, which involves both the *trnH* and *rps19* genes, represents a typical conserved feature that has evolved in the chloroplast genomes of angiosperms [50]. Crucially, the JSA/IRa boundary analysis reveals that the Guangxi accession exhibits a unique distance of 1172 bp for the *ycf1* fragment, which differs from the Fujian (1181 bp) and Yunnan (1181 bp) accessions. These boundary fluctuations, visualized in the IR plot, underscore the genetic divergence of the Guangxi ‘daodi’ (genuine) germplasm from other regional populations, suggesting potential adaptations to the specific Karst topography [51,52] and humid subtropical climate of the Lingnan region, but the present data do not establish that the boundary configuration is caused by karst adaptation or that it is fixed across the Guangxi population. Overall, the IR boundary structures of *A. verus* from Guangxi are more similar to those of *A. verus* and *A. gramineus*, aligning with their phylogenetic relationships. To identify highly variable hotspots within the genus and evaluate their potential as molecular markers, a genome-wide collinearity alignment was conducted.

The mVISTA and sliding-window results further show that sequence variation is concentrated in a small number of loci. The highest-diversity region was *psbM–trnD-GUC*, followed by *ndhF–rpl32* and several LSC intergenic or intronic regions. This uneven distribution is more informative for marker selection than the conserved majority of the plastome. These loci can be developed as candidate DNA barcodes or SNP/indel targets for species discrimination, provenance screening, germplasm traceability, and population-genetic surveys [53,54,55,56,57,58]. However, π was calculated across seven plastomes, with only one Guangxi accession. The hotspots should therefore be interpreted as comparative candidate regions, not as validated Guangxi-specific alleles. Pairwise testing across multiple Guangxi populations and matched Yunnan and Fujian samples, together with adulterants or closely related taxa, is required before these regions can be used for routine origin authentication.

The complete chloroplast genome sequence has emerged as a crucial resource for plant phylogenetic reconstruction and species classification, owing to its substantial information content, moderate evolutionary rate, and maternal uniparental inheritance [59,60]. The phylogenetic topology obtained in this study is generally consistent with previous studies of *Acorus*, which also recovered clear differentiation among the major taxa of the genus [61]. The ML and BI trees placed the Guangxi accession within the *A. verus* lineage and as sister to the Yunnan and Fujian accessions with strong support (BS/PP = 100/1). This topology supports the conspecific identity of the Guangxi sample and confirms close plastome affinity among the three regional accessions. It does not, by itself, prove a distinct geographic haplotype, a causal relationship with karst conditions, or a direct link between plastome variation and medicinal quality. The Guangxi plastome is therefore best viewed as a regional reference that enables the next stage of population-level and chemically informed germplasm evaluation.

Compared with the Yunnan and Fujian accessions, it has the shortest plastome, the highest GC content, and localized differences in LSC/IR length and IR boundary configuration, while retaining the conserved *Acorus* gene framework. The genome also contains candidate variable regions, particularly *psbM–trnD-GUC* and *ndhF–rpl32*, that may support future molecular identification and provenance studies. The scope of these conclusions is limited by the use of one Guangxi accession and by the maternal, single-linkage nature of chloroplast inheritance. The observed differences should therefore be treated as accession-level signals rather than province-wide characteristics. This study cannot determine whether the plastome features are stable across Guangxi populations, caused by karst environments, or associated with chemical quality. Future work should sample multiple *A. verus* populations and individuals across Guangxi, Yunnan, and Fujian; validate the candidate hotspots with pairwise SNP/indel assays; and integrate nuclear and mitochondrial markers with metabolomic and chemical-quality measurements. These tests will determine whether the candidate loci can distinguish provenance, track germplasm admixture, and support quality evaluation. Within its current scope, the Guangxi plastome provides a traceable molecular reference for conservation and for the sustainable development of this regional medicinal resource.

## 5. Conclusions

In this study, we analyzed the chloroplast whole genome of *A*. *verus* from Guangxi for the first time using high-throughput sequencing technology, elucidating its fundamental structural characteristics. The genome spans a total length of 152,784 bp and exhibits a typical tetrad ring structure, with a GC content of 38.74%. A total of 129 genes were annotated. Among the five reported samples of the genus *Acorus*, this genome is the shortest while exhibiting the highest GC content. Codon preference analysis indicated that the chloroplast genome of *A*. *verus* favors codons that end in A/T. Additionally, SSR analysis identified 89 microsatellite loci, predominantly single-nucleotide repeats (73%), which can serve as marker resources for the identification of germplasm and the evaluation of population genetic diversity. Comparative analysis of the IR region boundaries revealed that these boundaries are generally conserved across the seven samples of the genus *Acorus*. Notably, *ycf1* exhibited the greatest length variation and sequence divergence at the IRa/SSC boundary, suggesting its potential as a candidate barcode for molecular species identification. Phylogenetic analysis consistently placed Guangxi *A*. *verus* within the monophyletic branch of *A*. *verus*, thereby confirming its species affiliation at the molecular level.

In conclusion, the findings of this study enhance the genomic information resources of the genus *Acorus*, providing a crucial foundation for molecular identification, conservation of germplasm resources, and research on genetic diversity of local medicinal materials in Guangxi.

## Figures and Tables

**Figure 1:**
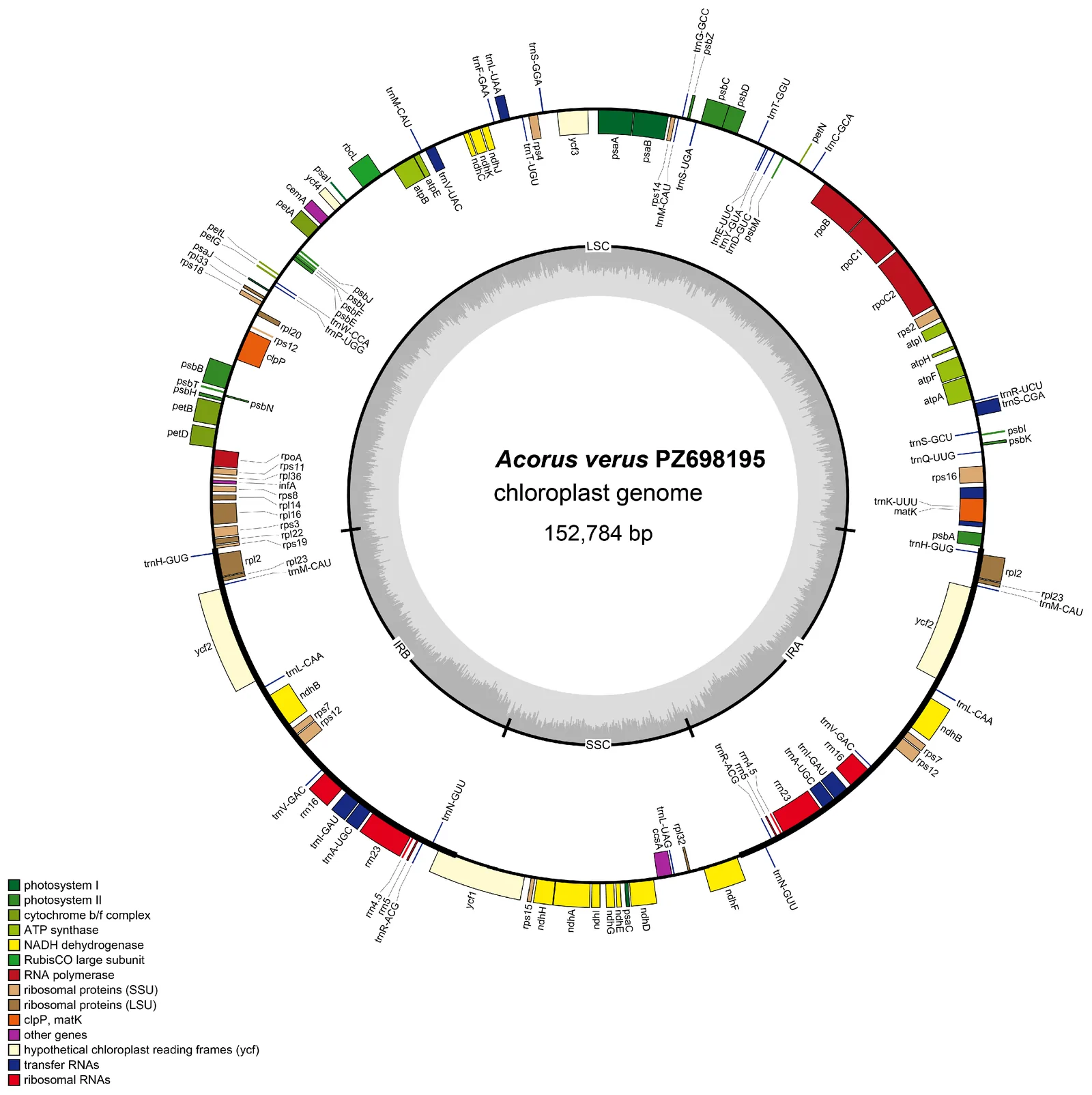
Chloroplast genome map of *A. verus* (PZ698195) from Guangxi, China.

**Figure 2:**
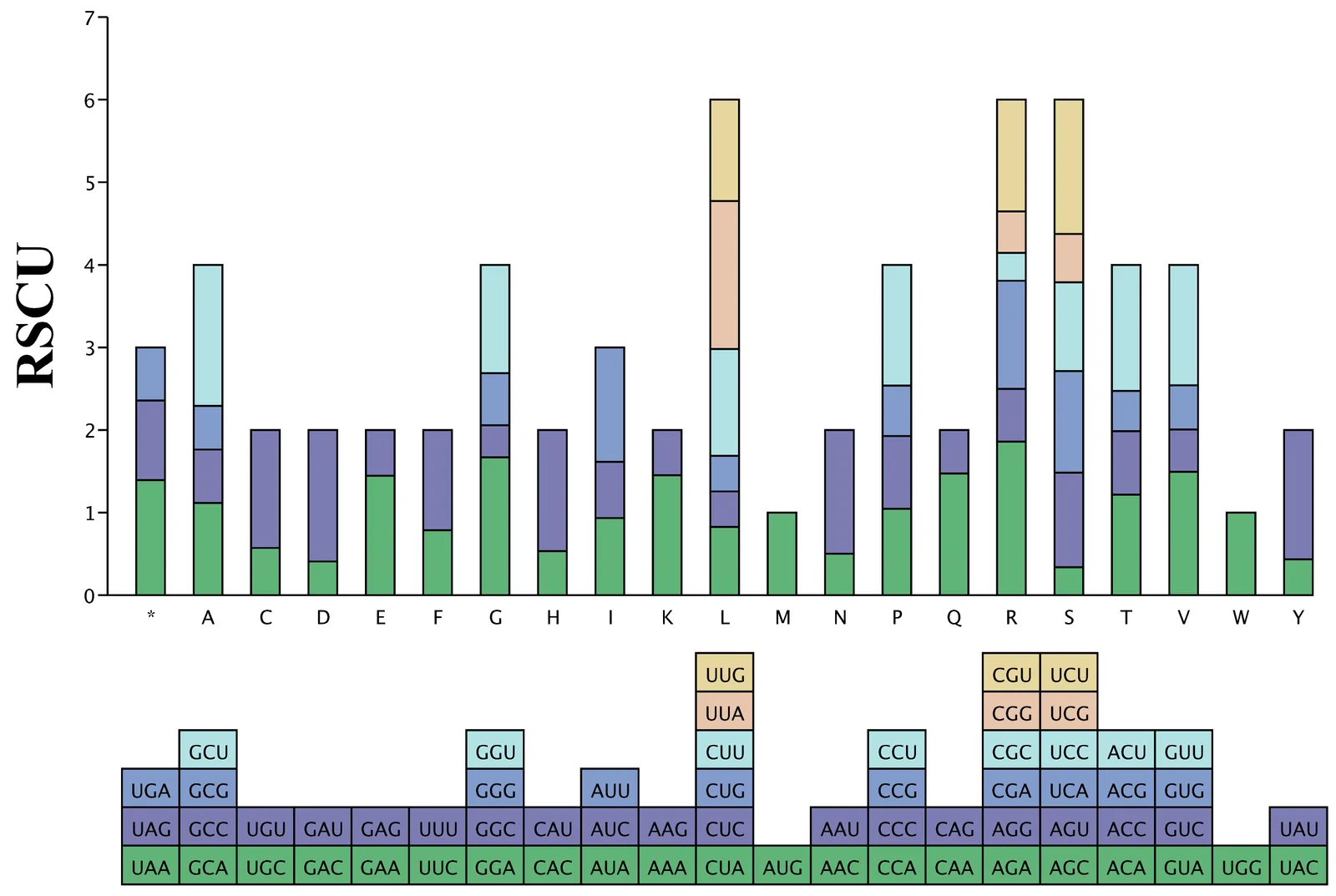
Codon RSCU statistical plot of the chloroplast genome of *A*. *verus* from Guangxi, China. “*” denotes a stop codon.

**Figure 3:**
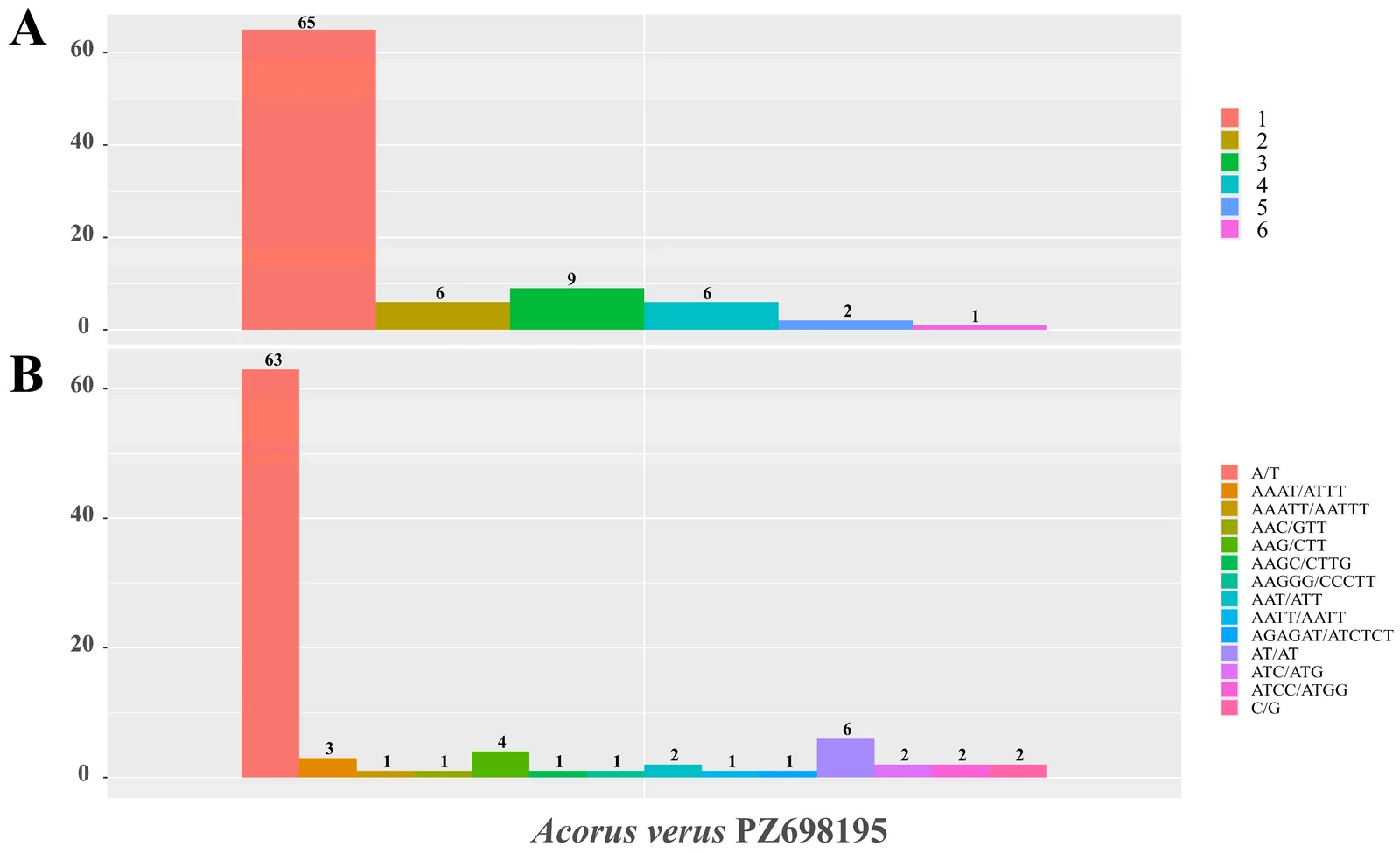
Types and quantities of simple sequence repeats in the chloroplast genome of *A*. *verus* from Guangxi. Note: In panel (**A**), the numbers 1, 2, 3, 4, 5, and 6 correspond to mononucleotide, dinucleotide, trinucleotide, tetranucleotide, pentanucleotide, and hexanucleotide repeats, respectively. (**B**) Number of SSR loci by motif; the color key identifies the motifs, and the values above the bars indicate the counts.

**Figure 4:**
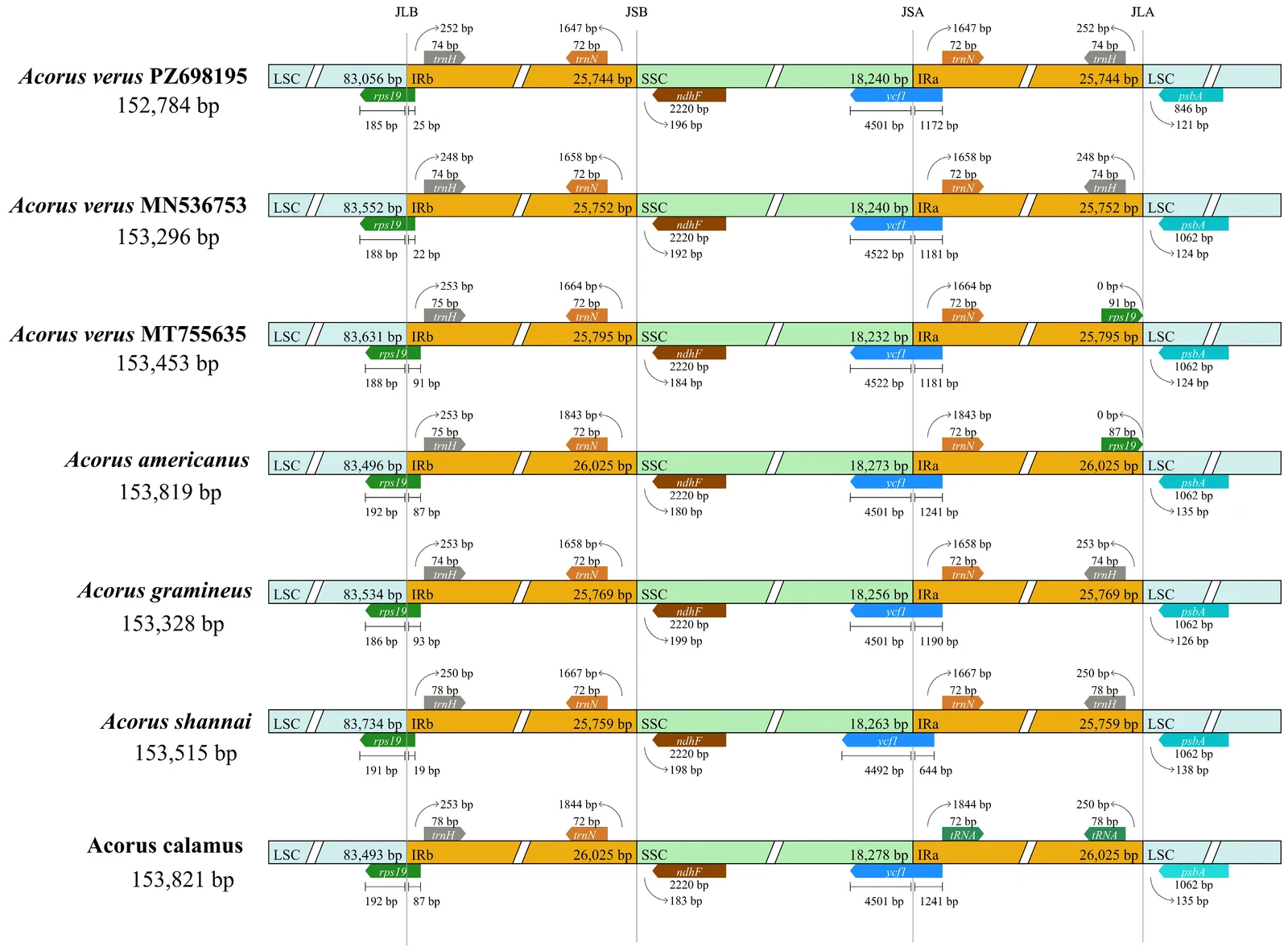
The boundary regions in the chloroplast genomes of *Acorus* species. Note: JLB (junction of LSC and IRb, the left junction between the large single-copy region and IRb), JSB (junction of SSC and IRb, the right junction between the small single-copy region and IRb), JLA (junction of SSC and IRa, the left junction between the small single-copy region and IRa), and JSA (junction of LSC and IRa, the right junction between the large single-copy region and IRa).

**Figure 5:**
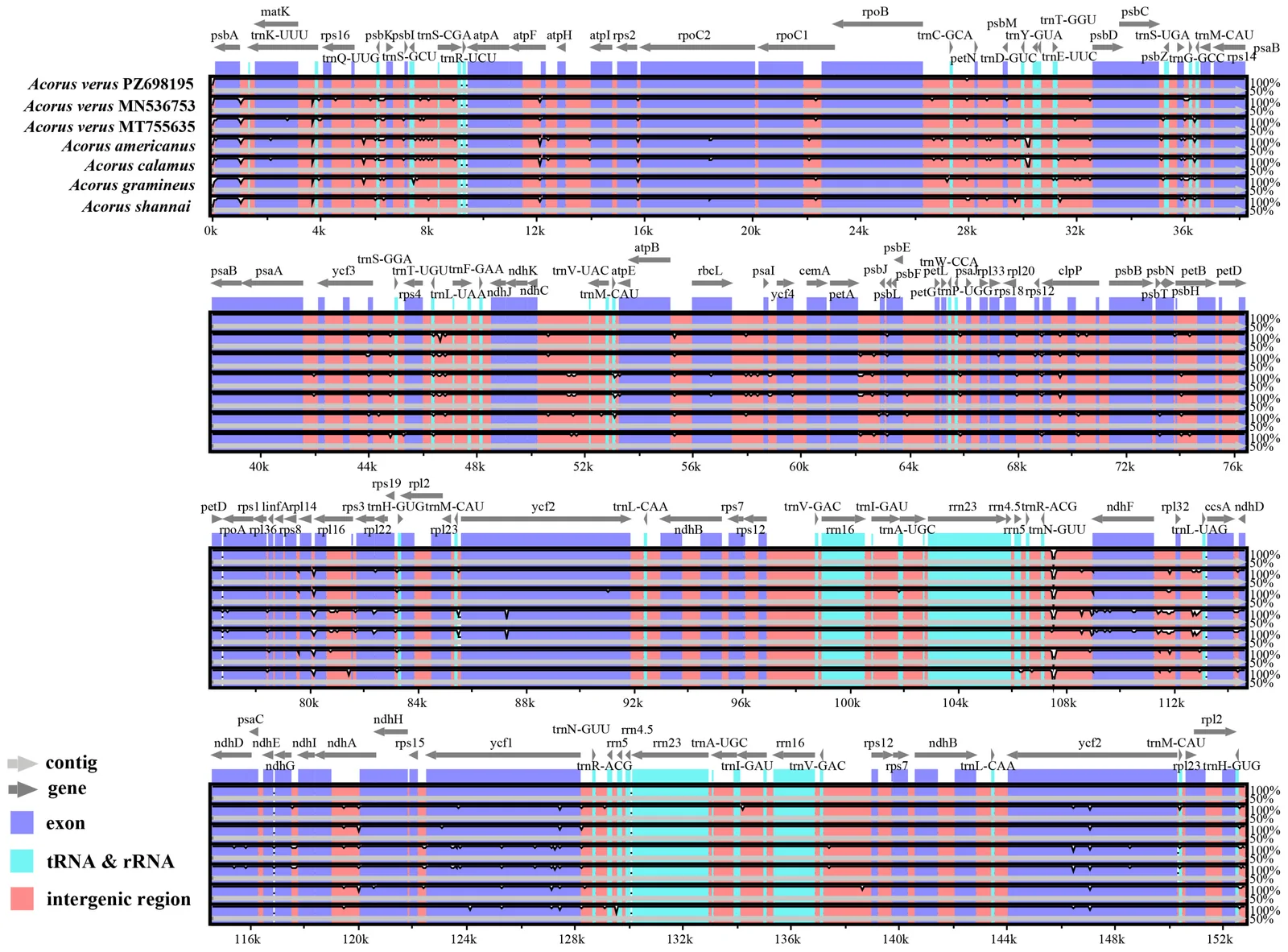
Analysis of chloroplast genome sequence similarity in *Acorus* species.

**Figure 6:**
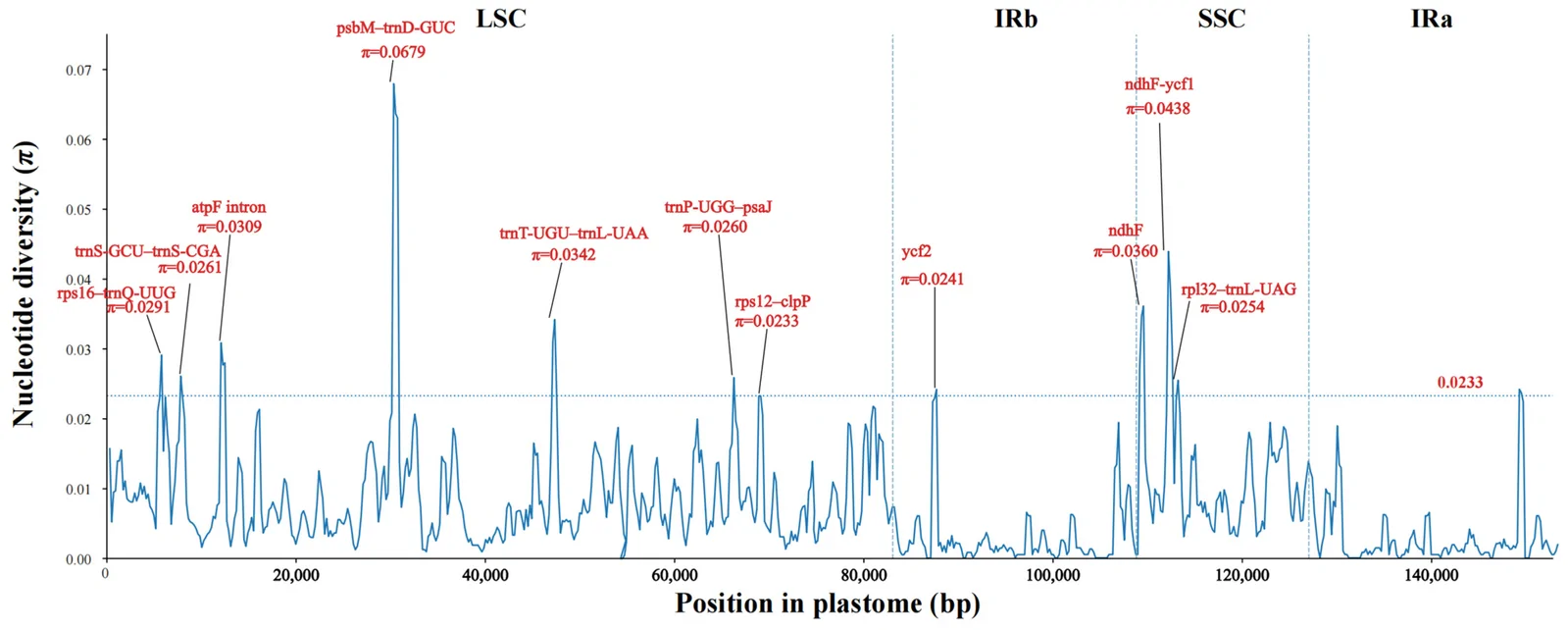
Sliding-window analysis of nucleotide diversity (π) among seven *Acorus* chloroplast genomes. Nucleotide diversity was calculated using a window length of 600 bp and a step size of 200 bp. Genome positions are based on the *A. verus* PZ698195 plastome. The LSC, IRb, SSC, and IRa regions are indicated, and regions with relatively elevated nucleotide diversity are labeled.

**Figure 7:**
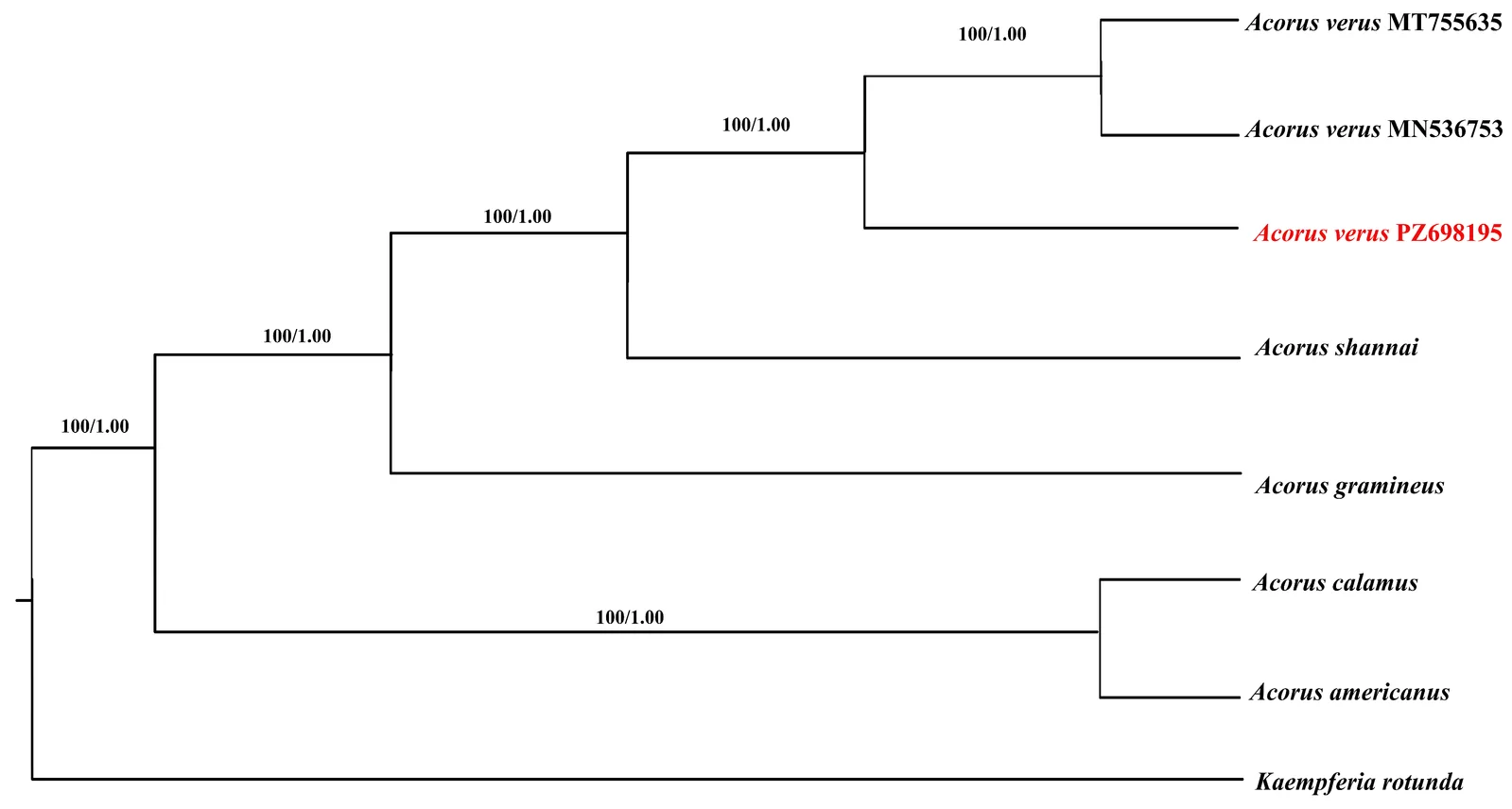
Phylogenetic relationships among selected *Acorus* plastomes inferred using maximum-likelihood (ML) and Bayesian inference (BI) analyses. Numbers at the nodes indicate ML bootstrap support values and Bayesian posterior probabilities (BS/PP), respectively. The Guangxi accession generated in this study is highlighted in red.

**Table 1:** Chloroplast genome gene content and features of *Acorus*.

Gene Functional Classification	Gene Group	Gene Name
Photosynthesis-related genes	Subunits of photosystem I	*psaA*, *psaB*, *psaC*, *psaI*, *psaJ*
	Subunits of photosystem II	*psbA*, *psbB*, *psbC*, *psbD*, *psbE*, *psbF*, *psbH*, *psbI*, *psbJ*, *psbK*, *psbL*, *psbM*, *psbN*, *psbT*, *psbZ*
	Cytochrome b/f complex	*petA*, *petB* *, *petD* *, *petG*, *petL*, *petN*
	Subunits of ATP synthase	*atpA*, *atpB*, *atpE*, *atpF* *, *atpH*, *atpI*
	Subunits of NADH dehydrogenase	*ndhA* *, *ndhB* *(2), *ndhC*, *ndhD*, *ndhE*, *ndhF*, *ndhG*, *ndhH*, *ndhI*, *ndhJ*, *ndhK*
	Large subunit of Rubisco	*rbcL*
Self-replicating related genes	Subunits of RNA polymerase	*rpoA*, *rpoB*, *rpoC1* *, *rpoC2*
	Proteins of large ribosomal subunits	*rpl14*, *rpl16* *, *rpl2* *(2), *rpl20*, *rpl22*, *rpl23* (2), *rpl32*, *rpl33*, *rpl36*
	Proteins of small ribosomal subunits	*rps11*, *rps12* **, *rps14*, *rps15*, *rps16* *, *rps18*, *rps19*, *rps2*, *rps3*, *rps4*, *rps7* (2), *rps8*
	Ribosomal RNAs	*rrn16S* (2), *rrn23S* (2), *rrn4.5S* (2), *rrn5S* (2)
	Transfer RNAs	*trnA-UGC* *(2), *trnC-GCA*, *trnD-GUC*, *trnE-UUC*, *trnF-GAA*, *trnG-GCC*, *trnH-GUG* (2), *trnI-GAU* *(2), *trnK-UUU* *, *trnL-CAA* (2), *trnL-UAA* *, *trnL-UAG*, *trnM-CAU* (4), *trnN-GUU* (2), *trnP-UGG*, *trnQ-UUG*, *trnR-ACG* (2), *trnR-UCU*, *trnS-CGA* *, *trnS-GCU*, *trnS-GGA*, *trnS-UGA*, *trnT-GGU*, *trnT-UGU*, *trnV-GAC* (2), *trnV-UAC* *, *trnW-CCA*, *trnY-GUA*
Other genes	Maturase gene	*matK*
	Protease gene	*clpP* **
	Envelope membrane protein gene	*cemA*
	C-type cytochrome synthesis gene	*ccsA*
	Translation initiation factor	*infA*
Unknown functional genes	Conserved open reading frames	*ycf1*, *ycf2* (2), *ycf3* **, *ycf4*

Note: “*” and “**” indicate that the gene has one and two introns, respectively; (2) and (4) indicate that the gene is duplicated two and four times.

**Table 2:** Taxonomic identities and accession information for the Acorus plastomes used in this study.

Accepted Taxon Name	Original Database Name	Accession	Geographic Origin	Original Publication
*Acorus americanus*	*Acorus calamus* var. *americanus*	EU273602	–	–
*Acorus calamus*	*Acorus calamus*	NC_007407	Jena, Germany	10.1093/molbev/msi173
*Acorus gramineus*	*Acorus gramineus*	OR855733	–	–
*Acorus shannai*	*Acorus shannai*	PQ456444	Guizhou, China	10.3897/phytokeys.251.139141
*Acorus verus*	*Acorus verus*	MN536753	Yunnan, China	10.1080/23802359.2019.1694852
*Acorus verus*	*Acorus calamus*	MT755635	Fujian, China	10.1080/23802359.2020.1806133
*Acorus verus* *	*Acorus verus*	PZ698195	Guangxi, China	–

Note: “*” The sample was collected from Guangxi, China (GenBank No. PZ698195).

**Table 3:** Characteristics of chloroplast genomes of verified *Acorus* taxa used in comparative analyses.

Accepted Taxon Name	Genome Length (bp)	LSC (bp)	SSC (bp)	IRs (bp)	GC Content (%)	Genes	CDS	tRNAs	rRNAs
*Acorus americanus*	153,819	83,496	18,273	26,025	38.59	132	84	38	8
*Acorus calamus*	153,821	84,149	18,278	25,697	38.6	132	86	38	8
*Acorus gramineus*	153,328	83,534	18,256	25,769	38.68	133	87	38	8
*Acorus shannai*	153,515	83,734	18,263	25,759	38.7	130	84	38	8
*Acorus verus*	153,296	83,532	18,240	25,752	38.7	132	86	38	8
*Acorus verus*	153,453	83,631	18,232	25,795	38.66	132	84	38	8
*Acorus verus* *	152,784	83,056	18,240	25,744	38.74	129	83	38	8

Note: “*” The sample was collected from Guangxi, China (GenBank No. PZ698195).

**Table 4:** Codon usage and relative synonymous codon usage (RSCU) values of *Acorus verus* PZ698195 in the analyzed genome.

Amino Acid	Codon	Count	RSCU	Amino Acid	Codon	Count	RSCU
Ter	UAA	26	1.3929	Met	AUG	540	1.0000
Ter	UAG	18	0.9643	Asn	AAC	275	0.5018
Ter	UGA	12	0.6429	Asn	AAU	821	1.4982
Ala	GCA	363	1.1178	Pro	CCA	263	1.0447
Ala	GCC	210	0.6467	Pro	CCC	222	0.8818
Ala	GCG	172	0.5296	Pro	CCG	154	0.6117
Ala	GCU	554	1.7059	Pro	CCU	368	1.4618
Cys	UGC	76	0.5714	Gln	CAA	629	1.4748
Cys	UGU	190	1.4286	Gln	CAG	224	0.5252
Asp	GAC	215	0.4087	Arg	AGA	453	1.8591
Asp	GAU	837	1.5913	Arg	AGG	156	0.6402
Glu	GAA	915	1.4478	Arg	CGA	319	1.3092
Glu	GAG	349	0.5522	Arg	CGC	82	0.3365
Phe	UUC	518	0.7884	Arg	CGG	122	0.5007
Phe	UUU	796	1.2116	Arg	CGU	330	1.3543
Gly	GGA	680	1.6697	Ser	AGC	105	0.3373
Gly	GGC	158	0.3880	Ser	AGU	357	1.1467
Gly	GGG	257	0.6311	Ser	UCA	383	1.2302
Gly	GGU	534	1.3112	Ser	UCC	334	1.0728
His	CAC	156	0.5324	Ser	UCG	183	0.5878
His	CAU	430	1.4676	Ser	UCU	506	1.6253
Ile	AUA	608	0.9354	Thr	ACA	371	1.2164
Ile	AUC	443	0.6815	Thr	ACC	235	0.7705
Ile	AUU	899	1.3831	Thr	ACG	149	0.4885
Lys	AAA	885	1.4532	Thr	ACU	465	1.5246
Lys	AAG	333	0.5468	Val	GUA	493	1.4951
Leu	CUA	346	0.8271	Val	GUC	169	0.5125
Leu	CUC	180	0.4303	Val	GUG	176	0.5337
Leu	CUG	180	0.4303	Val	GUU	481	1.4587
Leu	CUU	542	1.2956	Trp	UGG	428	1.0000
Leu	UUA	749	1.7904	Tyr	UAC	188	0.4362
Leu	UUG	513	1.2263	Tyr	UAU	674	1.5638
Ter	UAA	26	1.3929	Met	AUG	540	1.0000
Ter	UAG	18	0.9643	Asn	AAC	275	0.5018

## Data Availability

The datasets presented in this study can be found in online repositories. The names of the repository/repositories and accession number(s) can be found at https://www.ncbi.nlm.nih.gov/genbank/ (accessed on 16 July 2026), PZ698195.

## Supplementary Materials

The following supporting information can be downloaded at https://www.mdpi.com/article/10.3390/genes17101197/s1, Table S1: Summary of sequencing data quality statistics for the chloroplast genome of *Acorus verus* PZ698195.
