# Supplementary material for: Decoding Acorus verus: Chloroplast Genome Insights into a Key Medicinal Resource from Guangxi, Southern China
Source: Genes (Basel). 2026 Sep 27;17(10):1197. doi: 10.3390/genes17101197 (PMC13704511; doi:10.3390/genes17101197)
Supplement: Supplementary file 1 [file genes-17-01197-s001.zip › genes-4483894-supplementary.pdf]

Table S1 Summary of sequencing data quality statistics for the chloroplast genome of *Acorus verus* PZ698195.

Sample	Raw Data					Clean Data				
Acorus verus	**Reads**	**Bases**	**Q20**	**Q30**	**GC %**	**Reads**	**Bases**	**Q20**	**Q30**	**GC %**
PZ698195	27623284	4125224576	97.6%	93.5%	41.7%	3245044	483836478	97.4%	93.1%	39.4%
